# Invasive Fungal Breakthrough Infections under Targeted Echinocandin Prophylaxis in High-Risk Liver Transplant Recipients

**DOI:** 10.3390/jof9020272

**Published:** 2023-02-18

**Authors:** Robert Breitkopf, Benedikt Treml, Thomas Senoner, Zoran Bukumirić, Sasa Rajsic

**Affiliations:** 1 Department of Anaesthesiology and Intensive Care Medicine, Medical University Innsbruck, 6020 Innsbruck, Austria; 2 Institute of Medical Statistics and Informatics, Faculty of Medicine, University of Belgrade, 11000 Belgrade, Serbia

**Keywords:** liver transplantation, targeted antimycotic prophylaxis, echinocandins, invasive fungal infections, breakthrough infections

## Abstract

Invasive fungal infections (IFIs) are frequent and outcome-relevant complications in the early postoperative period after orthotopic liver transplantation (OLT). Recent guidelines recommend targeted antimycotic prophylaxis (TAP) for high-risk liver transplant recipients (HR-LTRs). However, the choice of antimycotic agent is still a subject of discussion. Echinocandins are increasingly being used due to their advantageous safety profile and the increasing number of non-albicans *Candida* infections. However, the evidence justifying their use remains rather sparse. Recently published data on breakthrough IFI (b-IFI) raise concerns about echinocandin efficacy, especially in the case of intra-abdominal candidiasis (IAC), which is the most common infection site after OLT. In this retrospective study, we analyzed 100 adult HR-LTRs undergoing first-time OLT and receiving echinocandin prophylaxis between 2017 and 2020 in a tertiary university hospital. We found a breakthrough incidence of 16%, having a significant impact on postoperative complications, graft survival, and mortality. The reasons for this may be multifactorial. Among the pathogen-related factors, we identified the breakthrough of *Candida parapsilosis* in 11% of patients and one case of persistent IFI due to the development of a secondary echinocandin resistance of an IAC caused by *Candida glabrata*. Consequently, the efficacy of echinocandin prophylaxis in liver transplantation should be questioned. Further studies are necessary to clarify the matter of breakthrough infections under echinocandin prophylaxis.

## 1. Introduction

Invasive fungal infections (IFIs) have been identified as one of the major outcome-determining complications after liver transplantation [1,2,3,4]. Given the incidence of 5% to 42%, they are still associated with high mortality rates of up to 50% in the case of candidiasis and up to 90% in the case of aspergillosis, the two main mycotic pathogens [5,6,7,8,9,10,11]. Based on predefined risk factors, targeted antimycotic prophylaxis (TAP) is recommended for high-risk liver transplant recipients (HR-LTRs) [12,13,14,15,16,17,18]. However, a consensus on the ideal antimycotic agent is still missing [19,20,21,22,23,24].

The use of echinocandins is supported by the lower nephrotoxic side effects (compared to amphotericin B) and the trend of a progressively rising rate of non-albicans *Candida* infections, which are partly less susceptible to triazoles [13,18,25,26,27,28,29]. Established as a first-line treatment for invasive candidiasis (IC), echinocandins have demonstrated broad efficacy with low toxicity [7,22,30,31,32,33,34]. Due to their low interaction potential, echinocandins do not interfere with coadministered immunosuppression and require no dosage adaption in the case of renal replacement therapy [35,36,37,38,39,40,41].

Despite the increasing use of echinocandins, current evidence on their benefits is still sparse [42]. Based on the recently published definition of breakthrough IFI (b-IFI) by the Mycoses Study Group Education and Research Consortium (MSG-ERC) and the European Confederation of Medical Mycology (ECMM), this retrospective study aims to investigate the incidence and risk factors for b-IFI in adult HR-LTRs under targeted echinocandin prophylaxis.

## 2. Materials and Methods

### 2.1. Study Population and Data Acquisition

**Figure 1:**
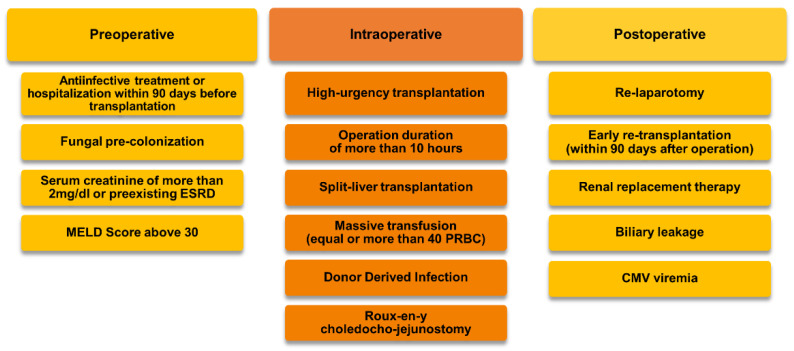
Perioperative risk factors for invasive fungal infections. Abbreviations: MELD: Model for End-Stage Liver Disease Score, ESRD: end-stage renal disease, PRBC: packed red blood cells, CMV: cytomegalovirus.

This retrospective study analyzed the risk of a breakthrough IFI (any IFI occurring during ongoing prophylaxis) in adult HR-LTRs managed with echinocandins as antifungal prophylaxis. We included all high-risk, first-time orthotopic liver transplant (OLT) recipients between January 2017 and December 2020 admitted to the intensive care units of the Department of Anesthesiology and Intensive Care Medicine of the Medical University of Innsbruck. Patients were classified as high risk for IFI if they had two or more (out of 15) predefined perioperative risk factors (Figure 1). Patients under 18 years of age, elective retransplantations more than 90 days after first transplant, combined organ transplantations, and patients receiving an active substance other than echinocandin monotherapy as antimycotic prophylaxis were excluded from our study. In the case of an early postoperative liver retransplantation (within 90 days after the first transplant), only data related to the first transplantation were analyzed.

We collected data on (1) patients’ sociodemographic characteristics, the underlying disease leading to OLT, Charlson Comorbidity Index, and the disease severity as measured by simplified acute physiology (SAPS III) and MELD score; (2) the transplantation procedure (organ donation, preservation, and implantation); (3) immunosuppression and anti-infective prophylaxis including the postoperative outcome, as well as graft and patient survival.

Moreover, we collected data on the incidence of fungal colonization, superficial and invasive fungal infection, agent and duration of prophylaxis, possible adverse events of prophylaxis, and perioperative risk factors for mycotic infection. All elective patients were screened for fungal precolonization at admission and were rated as colonized in the case of two or more positive clinical site surveillance cultures (nasal, pharyngeal, or rectal).

The date and the cause of death were extracted from the medical documentation. Death was attributed to IFI if there was a positive fungal culture or infectious process at the time of death, and no evidence of other causal factors was found.

Microbiological data were recorded for 90 days from the transplantation (end of study (EOS)), with long-term follow-up for patient and graft survival of 1 year after OLT. All positive microbiological findings were screened for contamination.

This work was approved by the Ethics Committee of the Medical University of Innsbruck, Austria (Number 1126/2022).

### 2.2. Definition of an Invasive Fungal Infection and Breakthrough Infection

A “proven” IFI was defined according to the definitions of the European Organization for Research and Treatment of Cancer/Invasive Fungal Infections Cooperative Group and the National Institute of Allergy and Infectious Diseases Mycoses Study Group (EORTC/MSG) Consensus Group [43]. Infections during ongoing critical care treatment were rated as “probable” upon the recommendations of the EORTC/MSGERC ICU Working Group [44].

Besides clinical symptoms of an infectious disease process, fundoscopic findings or hepatosplenic lesions by computed tomography were accepted as clinical criteria. Mycological criteria were defined by positive serum 1,3-β-d-glucan in two consecutive samples or recovery of Candida in an intra-abdominal specimen obtained surgically or within 24 h from external drainage. Candidemia was defined as the isolation of *Candida* spp. from at least one blood culture. An isolated Candida peritonitis without candidemia was rated as invasive only in cases with histopathologic or direct microscopic examination of perioperatively sampled sterile fluid or tissue. Positive samples taken from drains more than 24 h after surgery, as well as Candida isolation from respiratory secretions, stool, skin, and wound sites, and an asymptomatic candiduria were interpreted as colonization or “possible” infection in the case of clinical signs of sepsis [33,44,45,46,47].

Detection of *Aspergillus* spp. was only rated as “probable” invasive aspergillosis (IA) after positive mycological evidence of *Aspergillus* spp. either via cytology, direct microscopy, and/or culture in a lower respiratory tract specimen, or via a galactomannan antigen index >0.5 in plasma/serum, and/or galactomannan antigen >0.8 in bronchoalveolar lavage fluid (BALF) in the case of at least one diagnostic sign in computed tomography or bronchoscopic proof of a tracheobronchitis.

In the case of an IFI, computed tomography, transesophageal echocardiography, and fundoscopy were routinely performed to detect organ involvement.

Breakthrough IFI was defined as any IFI occurring during ongoing prophylaxis, including fungi outside its spectrum of activity. The time of b-IFI occurrence was defined as the first attributable clinical sign or symptom, mycological finding, or radiological feature. A relapse IFI was defined as occurrence after treatment and being caused by the same pathogen at the same site, although dissemination can occur [48].

### 2.3. Immunosuppression and Overall Anti-Infective Prophylaxis

The standard immunosuppression was performed by a triple combination of methylprednisolone, tacrolimus (target C0 levels of 7–10 ng/mL), and mycophenolate mofetil. In the case of tacrolimus-related side effects, it was switched to cyclosporine A (target C0 level of 150–200 ng/mL). Mycophenolate mofetil was switched to enteric-coated mycophenolate sodium or azathioprine in the case of gastrointestinal side effects.

Elective recipients received preoperative selective digestive decontamination (nonabsorbable antibiotics and oral amphotericin B), followed by extended-spectrum perioperative antibacterial prophylaxis with piperacillin–tazobactam for five days. Levofloxacin was used alternatively for patients with allergy against β-lactam agents.

Patients with high risk for cytomegalovirus (CMV) infection (seropositive donors, seronegative recipients) received antiviral prophylaxis (valganciclovir) for 3–6 months. A pre-emptive approach based on a weekly polymerase chain reaction surveillance was followed otherwise, and therapy was started only after detection of CMV viremia above a lower limit of quantification greater than 250 IU/mL before clinical symptoms.

At least once per week a routine, microbiological screening was performed (including Candida surveillance cultures from swabs of the throat, perineum, and urine cultures). A more-often sampling was performed if indicated by the critical care specialist.

### 2.4. Antimycotic Prophylaxis and Treatment

The targeted antimycotic prophylaxis included micafungin until the end of March 2019. Thereafter, anidulafungin was used based on the overarching recommendation of the local drug commission. In the case of pre-existing fungal colonization with echinocandin-resistant *Candida* spp. or *Aspergillus* spp., the prophylaxis was switched to fluconazole, voriconazole, or liposomal amphotericin B, and the patient was consequently excluded from the study.

Antimycotic prophylaxis was started as soon as the criteria for HR-LTRs were fulfilled. If TAP was started on the day of operation, it was rated as “immediate”, and “delayed” when started during the postoperative course. We used micafungin in a dosage of 100 mg/d or a loading dose of anidulafungin (200 mg), followed by 100 mg/d, both given intravenously.

In the case of a diagnosed infection, we adapted the antifungal regime as follows: fluconazole (800 mg loading dose, 1200–1600 mg with a body mass index >30 kg/m^2^), followed by a maintenance dose of at least 400 mg (600–800 mg with body mass index >30 kg/m^2^). The dosage was furtherly adjusted according to the renal function or in the case of renal replacement therapy. Voriconazole was initiated by two loading doses of 6 mg/kg every 12 h, followed by a maintenance dose according to a weekly performed therapeutic drug monitoring. Isavuconazole was started with 200 mg every 8 h for two days, followed by a daily dose of 200 mg. Liposomal amphotericin B (L-AmB) was dosed at 3 mg/kg per day.

The echinocandin prophylaxis was carried out over a period of minimum 7 to 14 days, and prolonged in the case of a diagnosed IFI or on clinical decision by the intensivist. Reasons for discontinuation of the prophylaxis were the completion of prophylaxis at discharge or missing clinical signs of infection, death, or switch to the therapeutic regime in the case of diagnosed infection.

Echinocandin therapy was continued in the case of invasive candidiasis and a positive response to therapy. In the case of confirmation of a fungal pathogen outside the echinocandin’s spectrum of activity or if a salvage therapy was indicated by the treating clinician, a switch to another agent was performed.

Antimycotic susceptibility was assessed according to the breakpoints determined by the European Committee on Antimicrobial Susceptibility Testing Subcommittee on Antifungal Susceptibility Testing [49]. Given the limited testing of echinocandins as first-line therapy of invasive aspergillosis, voriconazole was used as a first-line agent for aspergillosis, and isavuconazole as an alternative in the case of voriconazole-caused side effects or a suspected mucormycosis. [50,51] Liposomal amphotericin B or combination therapy of different antifungal agents has been used as a last option in critically ill patients. Fluconazole was used as first-line therapy for invasive *Candida parapsilosis* infections [22,23,24,33,48,52,53].

The duration of treatment continued for at least 14 days after the time of the last negative blood culture in the case of candidemia or until all clinical signs and symptoms had resolved.

### 2.5. Surgical Technique

An orthotopic transplantation of a standard criteria donated (SCD) whole organ of a deceased donor after brain death (DBD) after static cold storage (SCS) was defined as standard OLT. The recipient hepatectomy was performed by retrohepatic caval resection without venovenous bypass and the biliary anastomosis by duct-to-duct reconstruction. Deviations from this standard OLT were recorded.

From February 2018, normothermic machine perfusion (NMP) was implemented on a routine basis for donations after circulatory determination of death (DCD) in the case of surgically highly complex recipients or high-risk patients or for logistic reasons in the case of limited operative resources) [54].

Extended criteria were defined according to the Eurotransplant Foundation rules (donor age >65 years, donor body mass index >30 kg/m^2^, ICU stay with ventilation >7 days, serum sodium >165 mmol/L, hepatic steatosis >40%, total bilirubin >3 mg/dL, aspartate aminotransferase (AST) >90 U/L, alanine aminotransferase (ALT) >105 U/land DCD) [55].

### 2.6. Outcomes

The primary outcome was the occurrence of a proven or probable b-IFI during ongoing exposure to echinocandin prophylaxis within 90 days after transplantation at EOS [43,44].

The secondary outcomes related to fungal infections included the rate of any IFI (i.e., persistent, refractory, and relapse IFI, as well as donor-derived infections), fungal susceptibility, fungal colonization at the time of admittance, adverse events of the antimycotic prophylaxis and superficial fungal infections at EOS. Moreover, we analyzed 90-day mortality, 1-year graft and patient survival, and ICU-related events (e.g., length of ICU stay, rate of postoperative dialysis, ICU mortality, etc.) and postoperative surgical complications (e.g., bile leakage, reoperations, retransplantations, etc.).

### 2.7. Statistical Analyses

We performed all statistical analyses using SPSS (Version 22.0. Released 2013, Armonk, NY, USA: IBM Corp.). A significance level of 0.05 was applied, and all statistical assessments were two-sided. Depending on the data normality and the type of variables, results are presented as mean with standard deviation, median (range, minimum–maximum), and frequency (percent). For parametric data, the independent samples *t*-test was used, and the Mann–Whitney U test for numeric and ordinal data with non-normal distribution. The Chi-square test and Fisher’s exact test were used to test differences between the nominal data (frequencies). We used the univariate Cox proportional hazards model to investigate the potential risk factors for b-IFI occurrence. Covariates with a significance level of *p* < 0.05 were included in a multivariate model.

## 3. Results

### 3.1. Patient Population and Risk Factors for b-IFI

**Figure 2:**
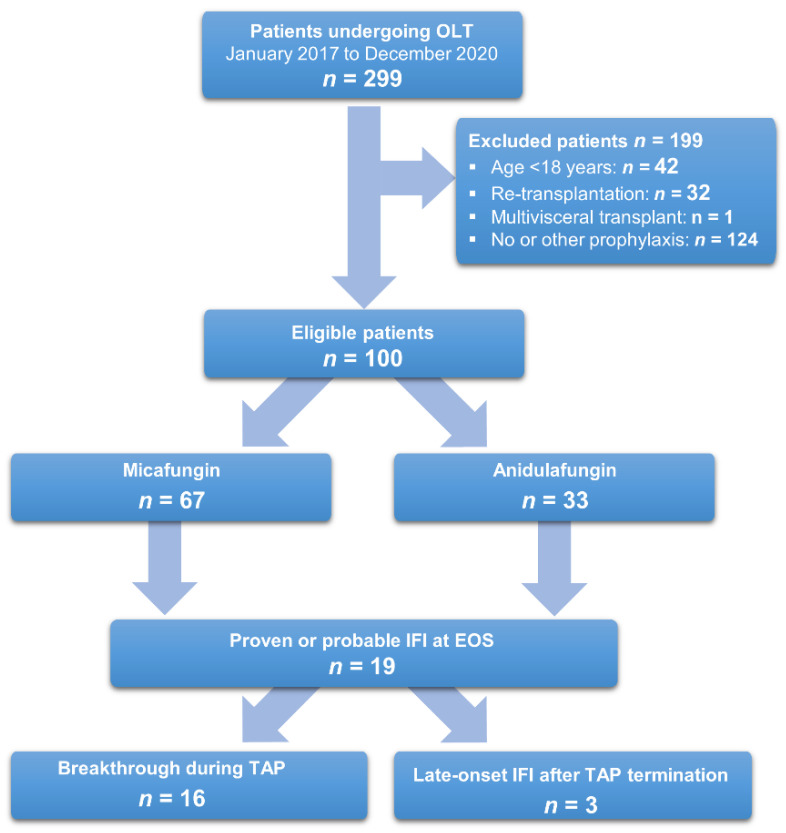
Flow chart of patient selection. Abbreviations: OLT: orthotopic liver transplantation; IFI: invasive fungal infection, b-IFI: breakthrough invasive fungal infection; EOS: end of study.

During the observation period, 299 patients underwent OLT, with 100 meeting the inclusion criteria (Figure 2).

The mean age was 57 ± 12 years with 75% (*n* = 75) male patients. Included patients had a mean SAPS III score of 46 ± 8, a median Charlson Comorbidity Index of 4 (0–10), and a median MELD score of 15 (6–40). Except for age, there were no significant differences between both groups of patients.

Cancers (33%, 33/100) and alcoholic cirrhosis (26%, 26/100) were the main underlying reasons for transplantation. Acute hepatic failure was diagnosed in 10% (10/100) of the cases.

The baseline demographics and clinical characteristics are summarized in Table 1.

Regarding the analyzed risk factors for IFI, there were no significant differences between the b-IFI and no IFI group of patients, apart from the type of donation (*p* = 0.028), the duration of cold ischemia time (*p* = 0.021), and the venous anastomosis (*p* = 0.024) (Table 1 and Table 2).

The univariate Cox regression analyses identified the age of the recipient, cold ischemia time (minutes), operative technique (piggyback), split-liver transplantation, donor-derived infection, bile leak, relaparotomy (any reason), and early retransplantation as independent risk factors for IFI development (Supplementary Table S1). Finally, recipient age and donor-derived infection had an increased hazard ratio for IFI within 90 days in the multivariate Cox regression model (Table 3).

### 3.2. Targeted Antimycotic Prophylaxis

Of the 224 patients undergoing first-time OLT, 100 (45%) received TAP with echinocandins, 67 (67%) patients received micafungin, and 33 (33%) patients received anidulafungin.

Within the group of patients with IFI, TAP was started immediately in 68% (13/19) of patients. In six patients (32%), TAP was started with a median delay of 13.5 days (11–55) due to the high-risk criteria not being met until the postoperative course. In most of the cases (83%, 5/6), the high-risk criteria were met first by the occurrence of biliary complications.

The overall median duration of prophylaxis was 9 (1–86) days, with no significant difference between the patients with and without b-IFI (8 (1–40) vs. 10 (1–49) days, *p* = 0.156). Within the group of patients with b-IFI, the duration of prophylaxis extended until the time of b-IFI diagnosis, since from this point onwards, antimycotic medication was regarded as therapy (Supplementary Table S2).

In 7% (7/100) of patients, drug-related adverse events were recorded (three cases of gastrointestinal symptoms, four cases with mildly elevated bilirubin and transaminase levels). All of these were associated with the usage of micafungin and limited only to the period of use. None of the adverse events were considered serious or dose-limiting.

### 3.3. Characteristics of Invasive Fungal Infections

#### 3.3.1. Incidence

Nineteen (19) (19%) patients developed a proven or probable IFI within 90 days after transplantation, with 16 (84%, 16/19) patients having a breakthrough infection. The rates of invasive fungal infections did not differ significantly between micafungin and anidulafungin (16%, 11/67 vs. 15%, 5/33, *p* = 1.000).

More than half of the IFIs (52%, 10/19) were diagnosed within the first two weeks after transplantation, and 68% (13/19) within the first month. Invasive candidiasis was diagnosed within a median of 13.5 days (3–77), and IA within 36 days (26–42). Almost one-third of patients (26%, 26/100) had been prehospitalized within three months before transplantation, and an associated fungal precolonization could be detected in 13% (13/100, *p* = 0.036) (Table 1).

#### 3.3.2. Composition of Pathogens and Infection Sites

Besides a single invasive *Saccharomyces cerevisiae* infection, two major pathogens dominated the fungal spectrum of the proven or probable infections: invasive candidiasis in 84% (16/19) and invasive aspergillosis in 16% (3/19) of patients (in one case as secondary fungal pathogen). Within the group of the non-albicans Candida species, in four cases, infections were caused by *C. glabrata* and in three by *C. krusei* (Table 4).

The main infection site in patients with an IC was isolated peritonitis without fungemia in 63% (10/16) of the patients, followed by peritonitis with secondary candidemia in 25% (4/16) and isolated candidemia in 13% (2/16) (Table 4). All cases of isolated candidemia were catheter related. The two cases of IA affected the lungs (without clinical proof of dissemination), and *Saccharomyces cerevisiae* caused isolated peritonitis. Eleven patients (11%) developed a superficial fungal infection (mucosal infection) during the observation period (Table 5). Finally, we could confirm three cases (16%, 3/19) of a donor-derived infection (Table 4).

#### 3.3.3. Susceptibility and the Therapeutic Regimen

Within the group of patients with IC, all cases of *Candida albicans* were susceptible to ongoing prophylaxis. In the case of non-albicans species, all cases of *Candida glabrata* were initially susceptible to anidulafungin (none received micafungin prophylaxis). One patient developed a refractory IFI due to secondary echinocandin resistance during ongoing therapy. Moreover, all cases of *Candida krusei* and *Candida dubliniensis* were tested as susceptible to echinocandins and echinocandins and triazoles, respectively. The sample of *Candida parapsilosis* was tested to be intermediately susceptible to anidulafungin (MIC 4 mg/L). Given the above, the detected primary pathogens were outside the spectrum of activity of echinocandin prophylaxis in 37.5% (6/16) of patients with b-IFI.

Within the patients with fungal organisms not susceptible to echinocandins, two patients died before an adaption of therapy could be performed. In the rest of the patients, the therapy was switched to voriconazole (*n* = 2), isavuconazole (*n* = 1), and a combination therapy of fluconazole and L-AmB (*n* = 1). Finally, in one patient, voriconazole was further escalated to L-AmB during the treatment.

Within the group of echinocandin-susceptible IFI patients, the antifungal agent was switched three times empirically to voriconazole; two of them were further escalated to L-AmB. In the remaining nine patients with proven b-IFI, the already-used echinocandin was prolonged until the end of therapy.

Finally, in addition to the case of proven secondary IA, two further cases of late-onset IFI (*Candida glabrata*) after the termination of TAP were identified. In both patients, IFI was diagnosed after an immediately started anidulafungin prophylaxis for over 17 and 20 days, with an onset of 60 and 24 days, respectively. In both cases, treatment with anidulafungin was resumed. During the study period, no case of relapse IFI was detected.

### 3.4. Outcome

#### 3.4.1. Postoperative Course

The median duration of the initial postoperative ICU stay was 7 (2–117) days (Table 5). More than one-half of patients (61%) needed renal replacement therapy, and more than one-third (37%) developed a CMV viremia. Surgical revisions were necessary for the majority of patients (54%), mostly due to biliary leakage (29%) and bleeding complications; 6% needed an early retransplantation.

Patients with b-IFI experienced an increased rate of biliary leaks (69% vs. 23%, *p* = 0.001) and more early retransplantations (19% vs. 4%, *p* = 0.050). The ICU mortality of these patients was 38%, being higher as compared to patients without b-IFI (7%, *p* = 0.003). The need for postoperative dialysis or occurrence of CMV viremia was comparable between both groups (Table 5).

#### 3.4.2. Mortality

Overall 90-day mortality was 11% (11/100). In the group without b-IFI (*n* = 7), two patients died due to bacterial sepsis, and one each due to intracerebral hemorrhage, postoperative hemorrhage, fungal sepsis, and acute liver failure (primary nonfunction). All patients died during their initial ICU stay, while one died intraoperatively during the transplantation.

**Figure 3:**
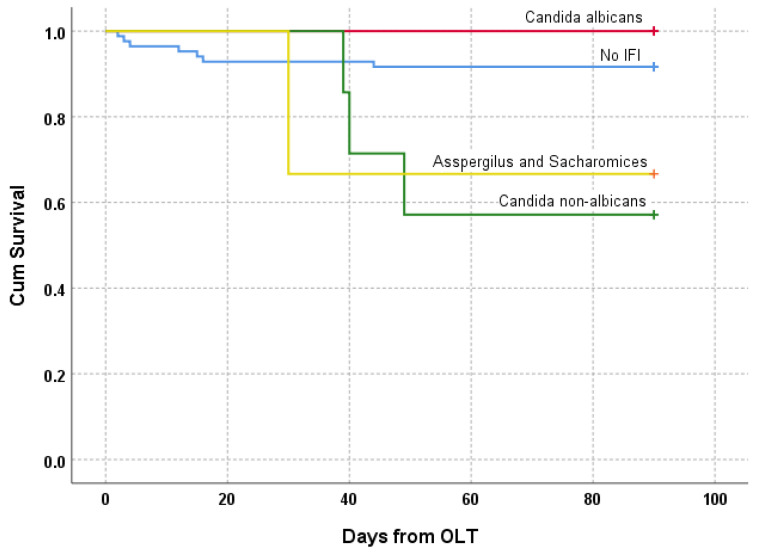
Kaplan–Meier curve: Mortality within 90 days depending on the cause of death. Abbreviations: OLT: orthotopic liver transplantation; IFI: invasive fungal infection.

Within the patients with b-IFI, 25% (4/16) died during the first 90 days after transplantation. All deaths were attributable to sepsis during an ongoing IFI and occurred after a median of 40 days (30–49) after OLT. Considering the causative pathogen, three out of four of the fatal IFIs were caused by non-albicans *Candida* spp. (*Candida krusei* and *Candida parapsilosis*), and one by *Aspergillus* spp. *(*Figure 3). All postoperative outcomes are summarized in Table 4 and Table 5.

Finally, the vast majority of patients (81%, 81/100) were alive one year after transplantation. However, in patients with b-IFI, one-year survival was significantly reduced to 50% (8/16 vs. 73/84, 87%; *p* = 0.002), and especially within the IC with non-albicans species, as 60% of patients died (Table 4). Moreover, none of the patients with IA survived the initial ICU stay, while five out of six (83%) patients with invasive *Candida albicans* infections survived.

## 4. Discussion

In this retrospective study, we present data from 100 adult patients undergoing first-time OLT with a high-risk profile for developing IFIs. In these patients, receiving targeted antimycotic prophylaxis with micafungin or anidulafungin, b-IFI was observed in 16%. Moreover, the overall incidence of IFIs within 90 days after transplantation was 19%. Recipient age and donor-derived infection were identified as the main risk factors for b-IFI. The observed incidence of b-IFI is rather high when compared to the international data (incidence up to 11%, Supplementary Table S3). This may be explained by the high rate of ECD organs (75%) and the main surgical technique of implantation (bicaval resection under transient clamping of the vena cava in 94% of the patients). Moreover, these factors could be a further reason for the high rate of reoperations (54%) and postoperative renal replacement therapy (61%), both possible risk factors and consequences of an IFI. Likewise, the high share of preoperative hospitalizations, the associated precolonization upon admission, and the rate of donor-derived infections may be further important factors. However, the available publications reported on rather smaller patient samples, which could lead to an underrepresentation of IFIs.

One-third of the primarily detected fungal pathogens were outside the spectrum of activity of the utilized echinocandin prophylaxis. This included three cases with IA (16%), and two cases with *Candida parapsilosis* (11%), which has been recently pointed out by the authors of the TRANSNET study [56].

Moreover, we detected one case of *Candida glabrata*, with a refractory IFI due to a secondary echinocandin resistance during ongoing therapy, but no proof of relapse IFI. This goes along with recent findings describing intra-abdominal candidiasis, particularly cases of *Candida glabrata*, as a possible hidden reservoir for the development of echinocandin resistance [57].

In this context, the importance of infection sites should be mentioned, since we could confirm reported data on intra-abdominal candidiasis being the main infection site after OLT [13,58,59]. In our population, nearly two-thirds of the IC patients developed isolated peritonitis without fungemia, followed by peritonitis with secondary candidemia in 25%. These findings are important as recently published data indicated that echinocandins might have limited penetration in the abdominal sites of infection [60,61]. Among the two cases of isolated candidemia, both were identified as fungal biofilm infections of vascular indwelling catheters, which were immediately removed at the time of diagnosis.

Although all cases of invasive *Candida albicans* infection would have been sensitive to fluconazole, the majority of the IC infections were caused by non-albicans species (63), of which only three would have been susceptible to fluconazole.

In regard to the adverse events of echinocandin prophylaxis, side effects were rarely observed. However, this could be also underrepresented given the retrospective nature of the study. In all seven cases, adverse events were described as being mild and reversible upon drug cessation.

Finally, more than one-half of the IFIs were diagnosed within the first two weeks after transplantation, with more than two-thirds within the first month, clearly demonstrating the importance of IFIs in the early postoperative course. The median time to diagnosis was depending on the pathogen, and was in compliance with the available literature (26–42).

Concerning clinical outcome, we could confirm one-year mortality associated with IFIs being nearly twice as high as without. Moreover, IFIs showed a significant impact on graft survival, especially during the early postoperative period. Invasive fungal infections were associated with a distinct increase in postoperative complications, resulting in an increased ICU mortality. All IFI patients, who died during their initial ICU stay, died directly from the fungal infection within two to three weeks.

## 5. Limitations

The presented study should be interpreted in light of the retrospective and monocentric study design limitations. A possible selection bias cannot be excluded. Despite the fact that the sample size is limited by the scope of this monocentric study, it represents one of the largest recently published studies in this field. Our results require further prospective confirmation to identify and account for unknown factors that might have an influence on the outcomes. Moreover, the attribution of IFIs on mortality is limited by possible effects of the underlying disease and the postoperative course.

## 6. Conclusions

Despite the profound evidence regarding risk factors in liver transplant recipients enabling the targeted use of antifungals as prophylaxis for IFIs, there is still a significant risk for b-IFI with a tremendous impact on patient outcome in the case of echinocandin use.

When selecting an antifungal therapy, toxicity, drug interactions, pharmacokinetic metabolism, and tissue penetration must be considered in addition to the antifungal spectrum, killing pattern, and clinical efficacy. While echinocandins are active and fungicidal against *Candida* spp., they are only fungistatic against *Aspergillus* spp. with no activity at all against most other molds.

Moreover, procedural, host, and fungal factors can contribute to a substantial rate of breakthrough infections. Our findings also raise doubts about the effectiveness of echinocandins in the case of intra-abdominal candidiasis, the most common site of fungal infection after OLT. Further prospective and randomized controlled trials are warranted to investigate the relationship between targeted echinocandin prophylaxis and b-IFI in HR-LTRs, providing clear recommendations on future perioperative management.

## Figures and Tables

**Table 1:** Sociodemographic and clinical characteristics, including the perioperative risk factors for b-IFI (*n* = 100).

Characteristics	All Patients (*n* = 100)	No b-IFI (*n* = 84)	b-IFI (*n* = 16)	*p*-Value
Age (years)	55.8 ± 12.1	57.4 ± 10.8	47.7 ± 14.0	0.003
Male sex	75 (75.0)	66 (78.6)	9 (56.3)	0.111
Weight (kg)	79 ± 18	80 ± 17	75 ± 21	0.290
Height (cm)	174 ± 9	174 ± 9	175 ± 9	0.759
Body mass index (kg/m^2^)	26.2 ± 5.5	26.5 ± 5.3	24.3 ± 6.0	0.150
SAPS III score	45.6 ± 8.1	45.8 ± 8.1	44.0 ± 8.4	0.458
MELD score	15 (6–40)	15 (6–40)	14.5 (9–40)	0.777
Charlson Comorbidity Index	4 (0–10)	4 (0–10)	4 (0–8)	0.980
**Underlying disease**				
Cirrhosis—Alcoholic cirrhosis	26 (26.0)	21 (25.0)	5 (31.3)	0.559
Malignancy and other tumors	33 (33.0)	29 (34.5)	4 (25.0)	
Cirrhosis—Virus related	5 (5.0)	4 (4.8)	1 (6.3)	
Cirrhosis—Nonalcoholic fatty liver cirrhosis	5 (5.0)	5 (6.0)	0 (0.0)	
Acute hepatic failure	10 (10.0)	8 (9.5)	2 (12.5)	
Cholestatic diseases	12 (12.0)	11 (13.1)	1 (6.3)	
Cirrhosis—Autoimmune cirrhosis	3 (3.0)	2 (2.4)	1 (6.3)	
Metabolic diseases	4 (4.0)	3 (3.6)	1 (6.3)	
Other	2 (2.0)	1 (1.2)	1 (6.3)	
**Risk Factors**				
MELD score >30	17 (17.0)	14 (16.7)	3 (18.8)	1.000
Fungal precolonization	13 (13.0)	10 (11.9)	3 (18.8)	0.432
Prehospitalization	26 (26.0)	24 (28.6)	2 (12.5)	0.227
SCr >2/ESRD	14 (14.0)	13 (15.5)	1 (6.3)	0.458
Organ donation type				
Whole organ donation	95 (95.0)	82 (97.6)	13 (81.3)	0.028
Split-liver donation	5 (5.0)	2 (2.4)	3 (18.8)	
High urgency transplantation	9 (9.0)	7 (8.3)	2 (12.5)	0.633
CMV status	53 (54.1)	47 (56.6)	6 (40.0)	0.270
High risk (D+/R-)	32 (34.8)	28 (36.4)	4 (26.7)	
Intermediate risk (D+/R+, D-/R+)	33 (35.9)	27 (35.1)	6 (40.0)	0.824
Low risk (D-/R-)	27 (29.3)	22 (28.6)	5 (33.3)	
Extended criteria donation	75 (75.0)	63 (75.0)	12 (75.0)	1.000
Donor age (years)	47.8 ± 16.1	48.6 ± 16.3	43.3 ± 15.1	0.229
Donation death type				
DBD	92 (92.0)	77 (91.7)	15 (93.8)	1.000
DCD	8 (8.0)	7 (8.3)	1 (6.3)	
Organ preservation				
Static cold storage	68 (68.0)	55 (65.5)	13 (81.3)	0.257
Normothermic machine perfusion	32 (32.0)	29 (34.5)	3 (18.8)	
Total operation time (minutes)	216 (175–754)	384 (175–754)	383 (188–614)	0.840
Cold ischemia time (minutes)	474 (171–1199)	470 (185–1199)	385 (171–724)	0.021
Prolonged operation (≥10 h)	2 (2.0)	2 (2.4)	0 (0.0)	1.000
Transfusion of ≥40 units PRBC	2 (2.0)	1 (1.2)	1 (6.3)	0.296
Venous anastomosis				
Retrocaval resection	94 (94.9)	82 (97.6)	12 (80.0)	0.024
Piggyback technique	5 (5.1)	2 (2.4)	3 (20.0)	
Biliary anastomosis				
Duct-to-duct reconstruction	90 (90.0)	76 (90.5)	14 (87.5)	0.660
Roux-en-Y choledochojejunostomy	10 (10.0)	8 (9.5)	2 (12.5)	

Data are presented as mean ± standard deviation, median (minimum–maximum range), or number of patients (%). Abbreviations: IFI: invasive fungal infection; b-IFI, breakthrough IFI; SAPS III: simplified acute physiology score III; MELD: Model for End-Stage Liver Disease; SCr: serum creatinine; ESRD: end-stage renal disease; DBD: donation after brain death; DCD: donation after circulatory death; CMV: cytomegalovirus; PRBC: packed red blood cells.

**Table 2:** Clinical characteristics and risk profile of patients with IFI (*n* = 19).

Patient Number	Sex	Ages (years)	Underlying Disease	Lab MELD Score	MELD Score >30	Fungal Precolonization	Prehospitalization	Serum Creatinin >2 mg/dL	Split-Liver Transplantation	HU Transplantation	ECD	DCD	NMP	Prolonged Operation	Massive Transfusion	RYC	Dialysis	CMV Viremia	Bile Leak	ERCP	Reconstruction	RYC	Relaparotomy, Other	Early Retransplantation
1	M	50	Cancers—Hepatocellular carcinoma and noncirrhotic liver	7							●						●		●			●		●
2	F	40	Acute hepatic failure	39	●		●			●	●						●	●	●			●		
3	M	61	Cancers—Hepatocellular carcinoma and cirrhosis	8							●						●	●					●	●
4	M	60	Cirrhosis—Alcoholic cirrhosis	13							●						●		●		●			
5	M	58	Cancers—Hepatocellular carcinoma and cirrhosis	13											●		●							
6	M	36	Cholestasis disease-Others: secondary sclerosing cholangitis	35	●	●	●	●			●						●		●		●			
7	F	24	Metabolic disease-Others: MNGIE	9					●							●	●	●	●		●			
8	F	57	Cirrhosis—Alcoholic cirrhosis	10					●							●			●		●			
9	F	22	Metabolic diseases-Wilson disease	22							●												●	
10	M	62	Cancers—Hepatocellular carcinoma and cirrhosis	11																				
11	F	57	Cirrhosis—Virus C-related cirrhosis	9							●								●		●			
12	M	67	Acute hepatic failure	40	●			●		●	●		●			●	●						●	
13	M	48	Cirrhosis—Alcoholic cirrhosis	13							●		●				●		●		●			
14	M	53	Cirrhosis—Alcoholic cirrhosis	18							●		●				●		●			●		
15	M	29	Cholestatic disease-Primary sclerosing cholangitis	25			●				●												●	
16	M	60	Cirrhosis—Alcoholic cirrhosis	22		●			●		●						●		●			●		
17	F	65	Acute hepatic failure	37	●					●	●						●						●	●
18	M	56	Cholestasis disease—Others: secondary biliary cirrhosis	17		●	●				●					●	●	●					●	●
19	F	55	Cirrhosis—Autoimmune cirrhosis	16		●					●	●	●				●	●	●		●			

Abbreviations: IFI: invasive fungal infections; M: male; F: female; MELD: Model for End-Stage Liver Disease Score; SCr: serum creatinine; HU: high urgency; ECD: extended criteria donation; DCD: donation after circulatory determination of death; NMP: normothermic machine perfusion; CMV: cytomegalovirus; ERCP: endoscopic retrograde cholangiopancreatography; RYC: Roux-en-Y choledochojejunostomy.

**Table 3:** Identification of risk factors for b-IFI: multivariate analysis (*n* = 100).

Nondependent Variable	B-Coefficient	*p*-Value	HR	95% Confidence Interval	
				Lower	Upper
Age (years)	−0.043	0.035	0.96	0.92	0.99
Cold ischemia time (minutes)	0.001	0.338	1.00	1.00	1.00
Piggyback operative technique	1.008	0.331	2.74	0.36	20.96
Split-liver transplantation	1.383	0.116	3.99	0.71	22.42
Donor-derived infection	2.478	0.003	11.92	2.35	60.30
Relaparotomy, any reason	1.016	0.127	2.76	0.75	10.19

Variables with increased hazard ratio for IFI: recipient age and donor-derived infection. Variables excluded from model (multicollinearity): bile leak and early transplantation. Abbreviations: IFI, invasive fungal infections; b-IFI, breakthrough IFI; HR, hazard ratio.

**Table 4:** Overview of the diagnosed IFI (*n* = 19).

Patient Number	Prophylaxis (Duration)	Fungal Pathogen	Type of Infection (Diagnosis day)	Definition of IFI	Donor-Derived Infection	Clinical Symptoms	Radiological Abnormalities	Histopathologic Examination	Culture Blood	Culture Catheter	PCR in Blood	Culture Abdominal Specimen	PCR in Abdominal Specimen	Culture BALF	PCR in BALF	Galactomannan in BALF	Fundoscopy	PCR Biopsy	Culture Urine	Therapeutic Regimen	Outcome
1	M (12)	*C. albicans*	Fungemia, Peritonitis (12)	P		●	●		●			●								1. Voriconazol 2. Amphotericin	Survived, graft failure d56, and Re-TX d58
2	M (12)	*C. krusei*	Fungemia, Peritonitis (12)	P		●	●		●		●	●							●	Anidulafungin	Death, sepsis at d39 (ICU)
3	M (3)	*C. albicans*	Peritonitis (3)	PR	●	●						●								Voriconazol	Survived, graft failure d2, Re-TX d2
4	M (1)	*C. albicans*	Peritonitis (14)	P		●	●	●				●								Micafungin	Survived
5	M (40)	*A. fumigatus*	Pneumonia (42)	P		●	●					●^1^		●		●			●	1. Voriconazol 2. Amphotericin	Death, sepsis at d110
6	M (8)	*C. dubliniensis*	Peritonitis (8)	P	●	●	●					●							●	1. Voriconazol 2. Amphotericin	Death, sepsis at d116
7	M (9)	*C. krusei*	Peritonitis (10)	P		●	●					●	●							Micafungin	Death, sepsis at d48 (hospital)
8	M (7)	*C. albicans*	Peritonitis (26)	P		●	●					●								Micafungin	Survived
9	M (1)	*C. albicans*	Peritonitis (4)	P	●	●	●					●								Micafungin	Survived
10	M (1)	*C. albicans*	Peritonitis (13)	PR		●						●								Micafungin	Death, sepsis at d221 (hospital)
11	M (1)	*Saccharomyces* spp.	Peritonitis (55)	P		●	●						●							Micafungin	Survived
12	A (14)	*C. glabrata*	Fungemia, Peritonitis (38)	P		●	●	●	●		●	●		●					●	Isovuconazol	Death, sepsis at d43 (ICU)
13	A (1)	*C. dubliniensis*	Peritonitis (37)	PR		●						●								Anidulafungin	Survived
14	A (21)	*C. parapsilosis*	Peritonitis (21)	P		●	●					●								Voriconazol	Death, sepsis at d38 (ICU)
15	A (20)	*C. glabrata*	Fungemia, catheter-related (44)	P		●			●	●										Anidulafungin	Graft failure d62, Re-TX d160. Death, sepsis at d329
16	A (13)	*C. krusei*	Peritonitis (13)	P		●	●					●								Anidulafungin	Survived
17	A (8)	*C. glabrata*	Fungemia, Peritonitis (8)	P		●	●		●			●							●	Fluconazol and Amphotericin	Graft failure d10, Re-TX d11. Death, sepsis at d91 (ICU)
18	A (17)	*C. glabrata*	Fungemia, catheter-related (77)	P		●			●	●									●	Anidulafungin	Survived, graft failure d2, Re-TX d4
19	A (15)	*A. fumigatus*	Pneumonia (26)	P		●	●	●			●			●	●	●				Anidulafungin	Death, sepsis at d26 (ICU)

^1^ Possible infection. Abbreviations: M: micafungin; A: anidulafungin; C.: Candida, A.: Aspergillus; IFI: invasive fungal infection, P: proven; PR: probable, d: day; Re-TX: retransplantation; ICU: intensive care unit.

**Table 5:** Postoperative outcome (*n* = 100).

Outcome	All Patients (*n* = 100)	No b-IFI (*n* = 84)	b-IFI (*n* = 16)	*p*-Value
Superficial fungal infections (mucosal)	11 (11.0)	11 (13.1)	0 (0.0)	0.207
Length of ICU stay (days)	7.0 (2–117)	6.5 (2–45)	18.0 (3–117)	0.054
Postoperative dialysis	61 (61.0)	49 (58.3)	12 (75.0)	0.270
Postoperative CMV viremia	37 (37.0)	33 (39.3)	4 (25.0)	0.399
Reoperations				
Bile leakage	30 (30.0)	19 (22.6)	11 (68.8)	0.001
Endoscopic retrograde cholangiopancreatography	1 (1.0)	1 (1.2)	0 (0.0)	1.000
Reconstruction	22 (22.0)	13 (15.5)	9 (56.3)	0.001
Secondary Roux-en-Y choledochojejunostomy	7 (7.0)	4 (4.8)	3 (18.8)	0.079
Early retransplantation (<90 days)	6 (6.0)	3 (3.6)	3 (18.8)	0.050
Other	29 (29.0)	26 (31.0)	3 (18.8)	0.385
Mortality within 90 days	11 (11.0)	7 (8.3)	4 (25.0)	0.073
Mortality during ICU stay	12 (12.0)	6 (7.1)	6 (37.5)	0.003
1-year Patient Survival	81 (81.0)	73 (86.9)	8 (50.0)	0.002
1-year Death-Censored Graft Survival	76 (93.8)	70 (95.9)	6 (75.0)	0.074

Data presented as median (minimum–maximum range) or number of patients (%). Abbreviations: IFI: invasive fungal infection; ICU: intensive care unit; CMV: cytomegalovirus; PRBC: packed red blood cells.

## Data Availability

The datasets used and analyzed during the current study can be made available from the corresponding author on reasonable request.

## Supplementary Materials

The following supporting information can be downloaded at: https://www.mdpi.com/article/10.3390/jof9020272/s1. Table S1. Risk factors for b-IFI within 90 days from liver transplantation: univariate Cox regression analyses (*n* = 100); Table S2. Antimycotic prophylaxis (*n* = 100); Table S3. Literature overview of Echinocandin use as prophylaxis in liver transplant recipients.
