# Supplementary material for: Invasive Fungal Breakthrough Infections under Targeted Echinocandin Prophylaxis in High-Risk Liver Transplant Recipients
Source: J Fungi (Basel). 2023 Feb 18;9(2):272. doi: 10.3390/jof9020272 (PMC9961099; doi:10.3390/jof9020272)
Supplement: Supplementary file 1 [file jof-09-00272-s001.zip › jof-2203794-supplementary-done.pdf]

Journal of *Fungi*

Invasive Fungal Breakthrough Infections under Targeted Echinocandin Prophylaxis in High-Risk Liver Transplant Recipients

Robert Breitkopf, Benedikt Treml, Thomas Senoner, Zoran Bukumirić and Sasa Rajsic

Supplementary material

Contents

Table S1. Risk factors for b-IFI within 90 days from liver transplantation: univariate Cox regression analyses (n = 100)
Table S2. Antimycotic prophylaxis (n = 100)
Table S3. Literature overview of Echinocandin use as prophylaxis in liver transplant recipients

Table S1. Risk factors for b-IFI within 90 days from liver transplantation: univariate Cox regression analyses (n = 100)

Nondependent variable	B-coefficient	P-value	HR	95% confidence interval		Missing data
				lower	upper	(n/Total)
Age (years)	-0.056	0.002	0.95	0.91	0.98	0/100
Sex (male)	0.973	0.054	2.65	0.99	7.11	0/100
Height (cm)	0.005	0.865	1.01	0.95	1.07	0/100
Weight (kg)	-0.018	0.225	0.98	0.95	1.01	0/100
Body mass index (kg/m2)	-0.077	0.126	0.93	0.84	1.02	0/100
SAPS III score	-0.022	0.530	0.98	0.91	1.05	0/100
MELD score	0.010	0.733	1.01	0.96	1.07	0/100
Charlson comorbidity index	-0.003	0.979	1.00	0.81	1.23	0/100
Cold ischemia time (minutes)	0.001	0.041	1.00	1.00	1.00	0/100
Warm ischemia time (minutes)	0.001	0.172	1.00	1.00	1.01	0/100
Surgery duration (minutes)	0.001	0.958	1.00	1.00	1.00	0/100
**Underlying disease malignancy and other tumors (reference category)**						0/100
Alcoholic liver disease	-1.768	0.115	0.17	0.02	1.54	
Virus related	-0.523	0.712	0.59	0.04	9.51	
Non-alcoholic fatty liver disease	-1.081	0.378	0.34	0.03	3.75	
Budd-Chiari syndrome	-	-	-	-	-	
Acute liver failure	-1.362	0.336	0.26	0.02	4.12	
Cholestatic	-	-	-	-	-	
Autoimmune hepatitis	-1.339	0.223	0.26	0.03	2.26	
Metabolic Liver Disease	-0.779	0.583	0.46	0.03	7.37	
Other	-2.116	0.135	0.12	0.01	1.93	
**Preoperative risk factors**						
MELD Score >30	0.270	0.674	1.31	0.37	4.60	0/100
Fungal colonization at baseline	0.486	0.449	1.63	0.46	5.71	0/100
Antiinfective pretreatment	-0.884	0.242	0.41	0.09	1.82	0/100
Pretransplant serum creatinine >2 mg/dl	-0.855	0.316	0.43	0.08	2.26	0/100
**Operative risk factors**						
Preservation (static cold storage)	-0.818	0.202	0.44	0.13	1.55	0/100
Choledochojejunostomy, primary	0.302	0.689	1.35	0.31	5.96	0/100
Piggyback operative technique	2.107	0.001	8.22	2.28	29.60	0/100
Transplantation time >11 hrs	-3.035	0.685	-	-	-	0/100
Intraoperative blood transfusion >40 PRBC	7.725	0.096	5.61	0.74	42.83	0/100
Split liver transplantation	1.700	0.008	5.45	1.54	19.25	0/100
Donor derived infection	2.124	0.001	8.36	2.37	29.56	0/100
High-urgency transplantation	0.574	0.448	1.78	0.40	7.82	0/100
CMV risk - high risk (D+/R-, reference category)						0/100
Intermediate risk (D+/R+, D-/R+)	-0.58	0.387	0.56	0.15	2.08	

Low risk (D-/R-)	-0.13	0.835	0.88	0.27	2.89	
Extended criteria donation	-0.019	0.973	0.98	0.32	3.04	0/100
Donation after circulatory determination of death	-0.289	0.780	0.75	0.10	5.67	0/100
**Postoperative risk factors**						
Bile leak	1.687	0.002	5.41	1.88	15.58	0/100
Relaparotomy, any reason	1.294	0.043	3.65	1.04	12.81	0/100
Early re-transplantation	1.669	0.009	5.31	1.51	18.70	0/100
Posttransplant dialysis	0.731	0.206	2.08	0.67	6.44	0/100
CMV viremia	-0.593	0.305	0.55	0.18	1.71	0/100

Abbreviations: IFI, invasive fungal infections; b-IFI, breakthrough IFI; SAPS III, simplified acute physiology score III; MELD, Model for End-Stage Liver Disease Score; PRBC, packed red blood cells; CMV, cytomegalovirus; HR, hazard ratio.

Table *S*2. Antimycotic prophylaxis (n = 100)

Antimycotic Prophylaxis	All patients (*n* =100)	No b-IFI (*n* =84)	b-IFI (*n* = 16)	P-value
Antimycotic agent				
Micafungin	67 (67.0)	56 (66.7)	11 (68.8)	1.000
Anidulafungin	33 (33.0)	28 (33.3)	5 (31.3)	
Duration of prophylaxis (days)	9 (1-86)	10 (1-49)	8 (1-40)	0.156

Data presented as number of patients (%). Abbreviations: IFI: invasive fungal infection; b-IFI, breakthrough IFI.

Table S3. Literature overview of Echinocandin use as prophylaxis in liver transplant recipients

Author	Agent	Duration of Prophylaxis (days)	n	90-days incidence
Doria et al. (2011) [59]	Caspofungin	8 (1-35)	16	0/16 (0)
Sun et al. (2013) [60]	Micafungin	20 (12-32)	18	2/18 (11.1)
Winston et al. (2014) [61]	Anidulafungin	21 (5-46)	98	5/98 (5.1)
Saliba et al. (2015) [62]	Micafungin	16.7 ± 7.0	172	4/172 (2.3)
Fortun et al. (2016) [63]	Caspofungin	22 (14-26)	97	4/97 (4.1)
Perella et al. (2016) [64]	Caspofungin	-	26	0/26 (0)
Neyra et al. (2020) [65]	Micafungin	12	33	1/33 (3)
Kang et al. (2020) [66]	Micafungin	20	68	1/68 (1.45)
Rinaldi et al. (2021) [67]	Caspofungin, Anidulafungin	13 (7-20)	95	9/95 (9.5)

Data presented as mean ± standard deviation, median (range) or number of patients (%).
